# Increased norovirus activity was associated with a novel norovirus GII.17 variant in Beijing, China during winter 2014–2015

**DOI:** 10.1186/s12879-015-1315-z

**Published:** 2015-12-18

**Authors:** Zhiyong Gao, Baiwei Liu, Da Huo, Hanqiu Yan, Lei Jia, Yiwei Du, Haikun Qian, Yang Yang, Xiaoli Wang, Jie Li, Quanyi Wang

**Affiliations:** Beijing Key Laboratory of Diagnostic and Traceability Technologies for Food Poisoning, Beijing Center for Disease Prevention and Control, No.16 Hepingli Middle Street, Dongcheng District Beijing, 100013 China

**Keywords:** Norovirus, Acute gastroenteritis, Outbreak, Genotype

## Abstract

**Background:**

Norovirus (NoV) is a leading cause of sporadic cases and outbreaks of acute gastroenteritis (AGE). Increased NoV activity was observed in Beijing, China during winter 2014–2015; therefore, we examined the epidemiological patterns and genetic characteristics of NoV in the sporadic cases and outbreaks.

**Methods:**

The weekly number of infectious diarrhea cases reported by all hospitals in Beijing was analyzed through the China information system for disease control and prevention. Fecal specimens were collected from the outbreaks and outpatients with AGE, and GI and GII NoVs were detected using real time reverse transcription polymerase chain reaction. The partial capsid genes and RNA-dependent RNA polymerase (RdRp) genes of NoV were both amplified and sequenced, and genotyping and phylogenetic analyses were performed.

**Results:**

Between December 2014 and March 2015, the number of infectious diarrhea cases in Beijing (10,626 cases) increased by 35.6 % over that of the previous year (7835 cases), and the detection rate of NoV (29.8 %, 191/640) among outpatients with AGE was significantly higher than in the previous year (12.9 %, 79/613) (*χ*^2^ = 53.252, *P* < 0.001). Between November 2014 and March 2015, 35 outbreaks of AGE were reported in Beijing, and NoVs were detected in 33 outbreaks, all of which belonged to the GII genogroup. NoVs were sequenced and genotyped in 22 outbreaks, among which 20 were caused by a novel GII.17 strain. Among outpatients with AGE, this novel GII.17 strain was first detected in an outpatient in August 2014, and it replaced GII.4 Sydney_2012 as the predominant variant between December 2014 and March 2015. A phylogenetic analysis of the capsid genes and RdRp genes revealed that this novel GII.17 strain was distinct from previously identified GII variants, and it was recently designated as GII.P17_GII.17. This variant was further clustered into two sub-groups, named GII.17_2012 and GII.17_2014. During winter 2014–2015, GII.17_2014 caused the majority of AGE outbreaks in China and Japan.

**Conclusions:**

During winter 2014–2015, a novel NoV GII.17 variant replaced the GII.4 variant Sydney 2012 as the predominant strain in Beijing, China and caused increased NoV activity.

## Background

Norovirus (NoV) is the most common cause of epidemic and sporadic cases of acute gastroenteritis (AGE) worldwide [1]. In the United States, a single suspected or confirmed etiology was implicated in 2819 AGE outbreaks during 2009 and 2010, and NoV was responsible for 1908 (68 %) outbreaks [2]. A systematic review of studies published between January 1, 2008 and March 8, 2014, estimated that global NoV prevalence in cases of AGE in community, outpatient, and inpatient settings was 24 % (95 % *CI* 18–30), 20 % (95 % *CI* 16–24), and 17 % (95 % *CI* 15–19), respectively [3].

The infectious dose for NoV is extremely low, with an estimated median infectious dose of 18 viruses [4]. NoV illness, which generally has an incubation of 24–48 h, is characterized by the acute onset of nausea, vomiting, abdominal cramps, and diarrhea, and these symptoms usually resolve in 2–3 days [5, 6]. NoV is mainly spread by oral–fecal contact through the ingestion of contaminated food or water or through direct contact with contaminated environmental surfaces or infected persons, but exposure to NoVs in air or in aerosolized vomitus has also been linked with infection [7].

NoV is a single-stranded, positive sense, RNA virus. Its genome ranges from 7.3 to 7.5 kb and is organized into three open reading frames (ORFs): ORF1 encodes a large polyprotein, which is cleaved into at least six mature non-structural proteins, including RNA-dependent RNA polymerase (RdRp); ORF2 and ORF3 encode the major (VP1) and minor (VP2) capsid proteins. The VP1 protein consists of three primary domains: the N-terminal domain (N), the highly conserved shell domain (S), and the protruding domain (P) that forms surface-exposed spikes. The P domain is subdivided further into the hypervariable P2 domain and the more conserved P1 domain; the protruding P2 domain possesses several epitopes that are involved in binding to the host cell [8]. NoV is classified into six genogroups (I–VI), and at least 39 ORF2-based genotypes have been described [7]. Only genogroups I, II, and IV have been found to infect humans, and genogroup II, genotype 4 (GII.4) strains are most commonly detected worldwide [9].

Between December 2014 and March 2015, an increased amount of infectious diarrhea cases was observed in Beijing, China, and the monthly detection rates of NoV among outpatients with AGE were higher than those of the previous year. Therefore, we examined the epidemiological patterns and genetic characteristics of NoV in sporadic cases and outbreaks.

## Methods

### AGE surveillance

The definition of infectious diarrhea used in this study was diarrhea (i.e., three or more loose stools within a 24 h period) and/or vomiting as the presenting symptom(s) of any disease caused by bacteria, viruses, fungi, or parasites; cholera, dysentery, typhoid, or paratyphoid were not included. The weekly numbers of infectious diarrhea cases between January 2012 and March 2015, which were reported by all hospitals in Beijing, were obtained from the China information system for disease control and prevention. These data was not freely available, and we had obtained the authorization from Beijing Center for Disease Prevention and Control (CDC). Cases of AGE were defined as patients who had diarrhea (as defined above) and/or vomiting symptoms. The new AGE outbreak surveillance was launched in April 2014, and an outbreak was defined as an occurrence of three or more cases of AGE within 3 days resulting from a common exposure. The outbreaks of AGE were reported by district-level CDCs, and fecal specimens were collected and detected. NoV-positive specimens were sent to the Beijing CDC, and 2–10 specimens per outbreak were randomly selected for sequencing. The monitoring system for sporadic AGE was launched in Beijing in 2011. Approximately 150 fecal specimens per month were collected from outpatients with AGE at the enteric disease clinics of 27 sentinel hospitals, which were located in different geographical regions across Beijing. The informed consent was obtained from the patients or their guardians for the stool samples.

### Viral RNA extraction

Viral RNA was extracted from 140 μL of a 10 % fecal suspension in phosphate-buffered saline using the QIAamp Viral RNA Mini Kit (QIAGEN, Hilden, Germany) according to the manufacturer’s protocol. Extracted RNA was stored at −20 °C until further use.

### NoV detection

GI and GII genogroup NoVs were detected using a SuperScript III Platinum® One-Step qRT-PCR Kit (Invitrogen, Carlsbad, CA, USA) with primers and probes as described previously [10].

### Reverse transcription polymerase chain reaction (RT-PCR)

The QIAGEN One-Step RT-PCR Kit (QIAGEN, Hilden, Germany) was used to amplify the genes of NoV in a 50-μL reaction volume. RNase Inhibitor (Promega, Madison, WI, USA) was added at a final concentration of 5–10 units/reaction. The revised primers 289/290 (p290, nt4568–4590, GATTACTCCAGGTGGGAYTCMAC; p289, nt4865–4886, TGACGATTTCATCATCMCCRTA; positions are indicated relative to the M87661 reference sequence) were used to amplify the partial RdRp gene of NoV (319 bp) [11]. RT-PCR was performed at 42 °C for 60 min and 95 °C for 15 min followed by 40 cycles of 94 °C for 30 s, 58 °C for 80 s, and 72 °C for 60 s; a final extension was run at 72 °C for 7 min. Primers G1SKF/G1SKR and G2SKF/G2SKR were used to amplify the partial VP1 genes of GI and GII NoVs, generating 330 bp and 344 bp PCR products [12]. RT-PCR was performed at 50 °C for 30 min and 95 °C for 15 min, followed by 40 cycles of 94 °C for 30 s, 55 °C for 30 s, and 72 °C for 60 s; a final extension was run at 72 °C for 7 min. The ORF1/ORF2 junction region (1095 bp) was amplified using primers 290 and G2SKR, and RT-PCR was performed under the same reaction conditions for the VP1 gene of NoV. The PCR products were analyzed using a QIAxcel Advanced Instrument with a QIAxcel DNA Screening Kit (QIAGEN, Hilden, Germany).

### DNA sequencing and phylogenetic analysis

PCR products were purified and sequenced directly on an ABI 3730×l DNA Analyzer using a BigDye Terminator v3.1 Cycle Sequencing Kit (ABI, Austin, TX, USA). All sequences were prepared and aligned by BioEdit (version 7.0.9.0) with the Clustal W program. Genotypes were determined by phylogenetic analyses with the Norovirus Typing Tool (available at http://www.rivm.nl/mpf/norovirus/typingtool). The phylogenetic tree was constructed using the maximum likelihood method with MEGA software (version 6.06) and bootstrap analysis was performed with 1000 replications. The sequences of the NoV strains detected in this study were deposited in GenBank (accession numbers KR095171-KR095172, KT634313-KT634314, and KT633382-KT633396).

### Statistical analysis

Statistical analyses were performed using the Statistical Package for Social Sciences (SPSS v13.0) software (SPSS Inc., Chicago, IL, USA), and statistical significance was set at *P* < 0.05.

### Ethical statement

This study was approved by the Ethics Committee of the Beijing CDC.

## Results

**Fig. 1:**
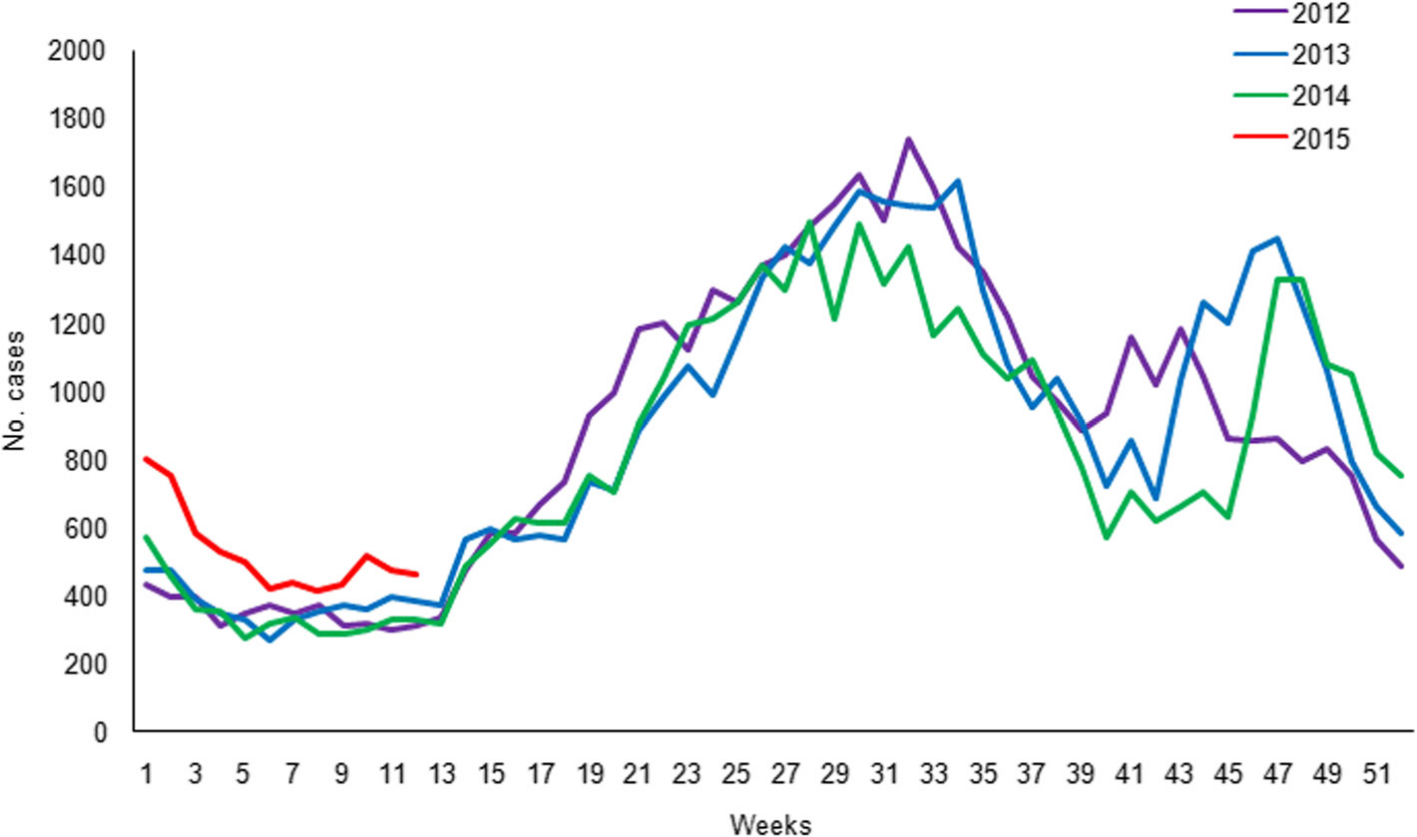
The weekly number of infectious diarrhea cases in Beijing, China, reported through China information system for disease control and prevention, between January 2012 and March 2015

**Fig. 2:**
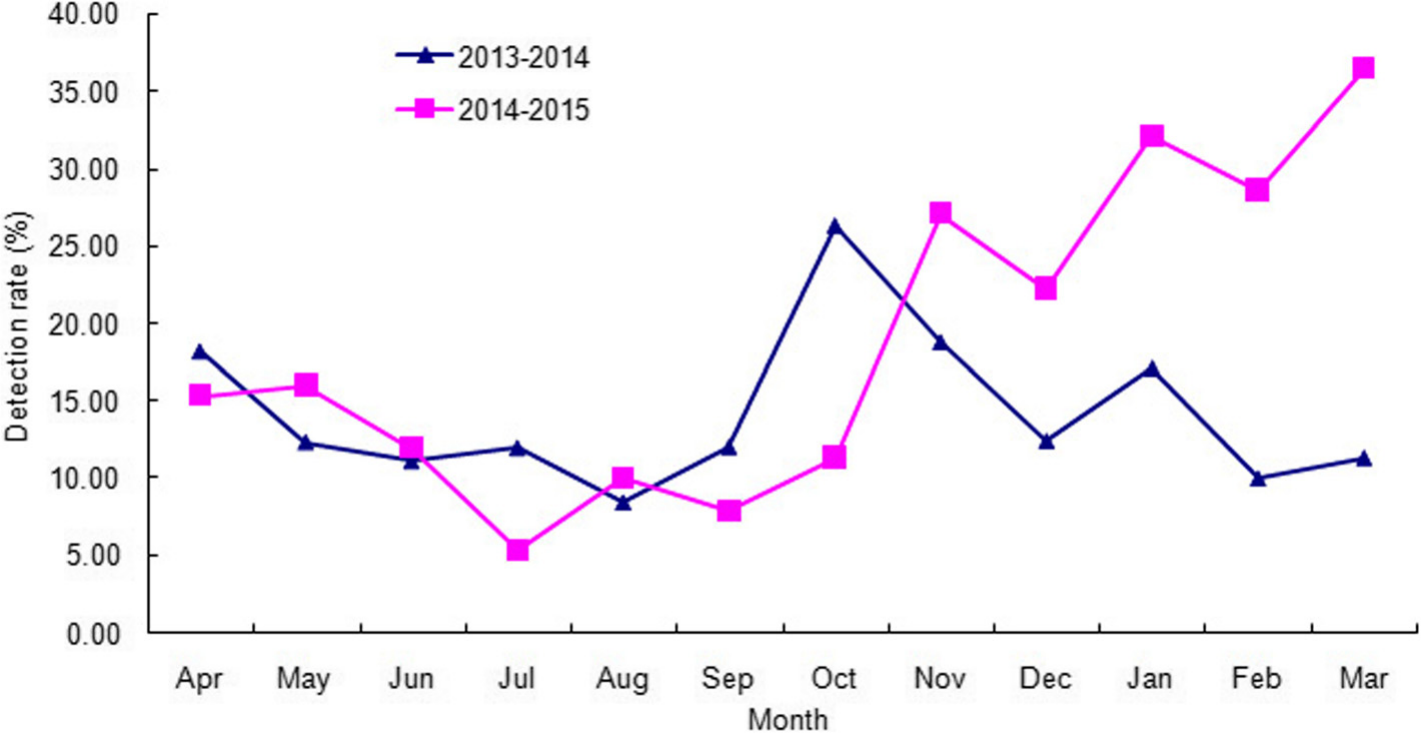
NoV detection rates by month between April 2013 and March 2015

**Fig. 3:**
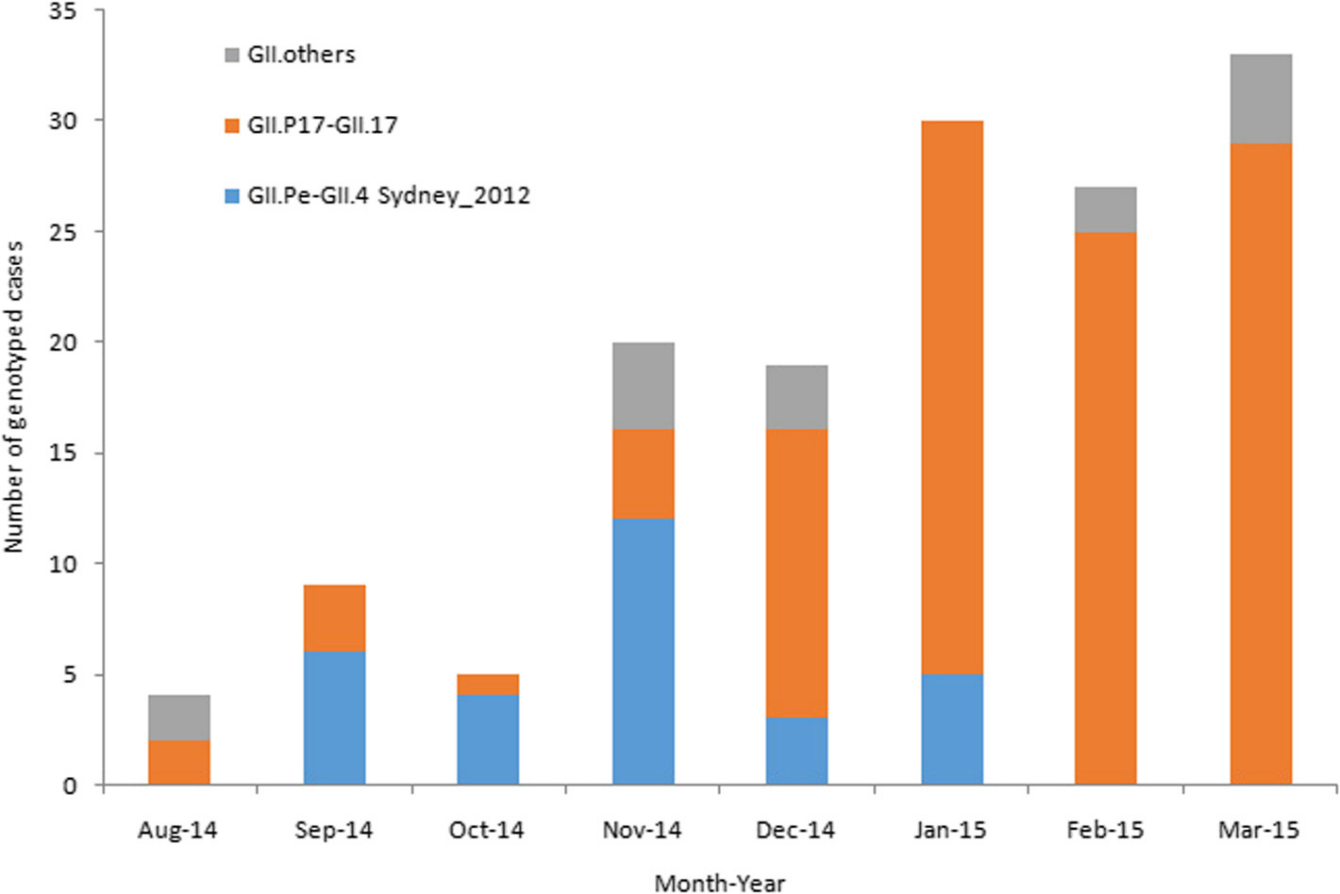
The monthly genotype distribution of GII NoVs among outpatients with acute gastroenteritis in Beijing, China, between August 2014 and March 2015

Between December 2014 and March 2015, the number of reported infectious diarrhea cases in Beijing (10,626 cases) increased by 35.6 % over that of the previous year (7835 cases; Fig. 1). During this period, the overall detection rate of NoV (29.8 %, 191/640) among outpatients with AGE was significantly higher than the overall rate from the previous year (12.9 %, 79/613) (*χ*^2^ = 53.252, *P* < 0.001), and the monthly detection rates of NoV among outpatients were also higher than those of the previous year (Fig. 2). Of the 191 NoV-positive specimens, 187 were identified to be NoV GII, 3 belonged to NoV GI, and one was a combined infection of NoV GI and GII. Of the 187 GII NoVs, 109 were sequenced and 92 (84.4 %) belonged to a novel GII.17 genotype. This novel GII.17 strain was first detected in an outpatient with AGE in August 2014, and it went on to replace GII.4 Sydney_2012 as the predominant variant between December 2014 and March 2015 (Fig. 3). The change in the detection rate of this GII.17 strain occurred concurrently with the increase in the number of reported infectious diarrhea cases in Beijing.

Between November 2014 and March 2015, 35 outbreaks of AGE were reported to the Beijing CDC, and NoVs were detected in 33 of these outbreaks, all of which belonged to the GII genogroup. The NoVs were sequenced and genotyped in 22 outbreaks, of which 20 were caused by this novel GII.17 strain (4/5 in November 2014, 4/4 in December 2014, 5/6 in January 2015, 2/2 in February 2015, and 5/5 in March 2015). Because the AGE outbreak surveillance was launched quite recently, the specimens from 11 outbreaks were not able to be obtained for sequencing. The first outbreak caused by this strain was reported at a university in October 2014, affecting 121 persons within 3 days, which was the only GII.17 outbreak reported that month.

**Fig. 4:**
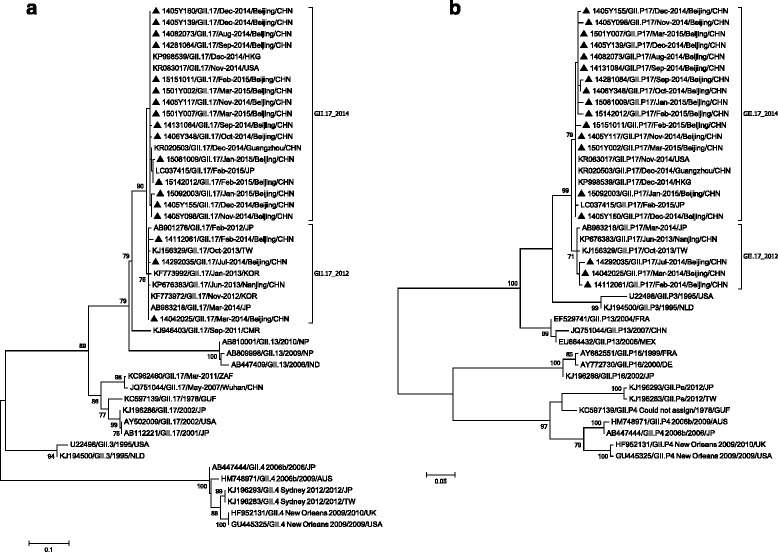
Phylogenetic analysis based on partial VP1 genes (282 bp) (**a**) and RdRp genes (274 bp) (**b**) of the GII.17 NoVs. NoV strains detected in this study were marked with the following symbols: ▲. The reference sequences were retrieved from GenBank. The trees were generated using the maximum likelihood method, nucleotide substitution models Kimura 2-parameter (K2) + Gamma (G) + Invariant sites (I) was used for VP1 genes and K2 + I for RdRp genes. Bootstrap values estimated with 1000 replicate data sets were indicated at each node. The scale bar indicated the number of nucleotide substitutions per site

A phylogenetic analysis of the NoV VP1 genes revealed that the new GII.17 strains formed a single cluster, sharing the highest identity with previous GII.17 strains (83.6–87.5 %) and with GII.13 strains (83.3–85.4 %) (Fig. 4a). The new GII.17 strains have existed mainly in eastern Asia since 2012, in contrast to previous GII.17 strains, which were prevalent worldwide up to 2011. The RdRp genes of this variant were analyzed, and the genotype could not be identified (Fig. 4b). The highest identity was shared with GII.P3 (85.3–89.7 %) and GII.P13 strains (84.9–88.6 %). The reference sequences of the GII.17 genotype were obtained from GenBank and were analyzed using the Norovirus Typing Tool. The most common genotype combination, based on VP1 and RdRp genes, was GII.P16_GII.17, and the other combinations included GII.P13_GII.17, GII.P4_GII.17, GII.Pe_GII.17, and GII.P3_GII.17. Because this was the first orphan ORF1 sequence associated with GII.17, it was designated GII.P17_ GII.17 in August 2014 [13]. The GII.P17_ GII.17 variant further clustered into two sub-groups in the VP1 and RdRp genes, named GII.17_2012 and GII.17_2014 according to the years in which they were first reported. The dominant strain in Beijing, Jiangsu, Guangdong, and Japan during winter 2014–2015 belonged to GII.17_2014.

## Discussion

Over the past two decades, NoV GII.4 strains have been responsible for the majority of both outbreaks and sporadic cases of AGE [1]. GII.4 variants have emerged every 2–3 years, and these new variants replaced the old ones as the predominant variant. The new variants have caused at least six major pandemics: US 95/96 (1995–1996), Farmington Hills (2002–2003), Hunter (2004–2005), Den-Haag_2006b (2006–2007), New Orleans_2009 (2009–2010), and Sydney_2012 (2012–2013) [14–19]. GII.3 was previously reported to be the most common non-GII.4 genotype in children (≤18 years of age) with AGE [20], and the other genotypes, such as GII.6 [21] and GII.13 [22, 23], also had higher detection rates in some regions in recent years, but none of these non-GII.4 genotypes have ever replaced the dominance of GII.4 genotypes.

Between December 2014 and March 2015, the detection rate of NoV among outpatients with AGE was significantly higher than that for the previous year, and GII.17_2014 was the dominant genotype during this time. Bacterial pathogens were rarely detected during these months, and there was no increase in the detection rate of group A rotavirus. Additionally, enteric adenoviruses and astroviruses were rare in Beijing during this study period (data not shown). This evidence indicates that GII.17_2014 caused the increase in the number of infectious diarrhea cases in Beijing between December 2014 and March 2015.

GII.4 Sydney_2012 was first detected in August 2012 and replaced Den Haag 2006b as the predominant variant in Beijing after September 2012. Between October and December 2012, the monthly detection rates of NoV were higher than those in the previous year, and the visits to enteric clinics increased by 44.6 % compared with the same period in the previous year [24]. Similar results were also observed with GII.17_2014 in this study, which suggests that this GII.17_2014 has the same epidemiological fitness in Beijing as GII.4 Sydney_2012.

There are limited data about GII.17 NoV worldwide. Earlier strains of GII.17 NoV were usually reported in sporadic cases of AGE with a low detection rate, and it was rarely linked with outbreaks [25–28]. Additionally, GII.17 NoV was also detected in ground water, wastewater, and river water [29–32]. Most of the GII.17 NoV sequences in GenBank only include partial VP1 genes, and very few sequences cover ORF1 and ORF2. The first report of GII.17_2012 in GenBank is from Japan in February 2012; thereafter, such sequences in GenBank are mainly from human specimens in East Asia (South Korea, China Mainland, and Taiwan) and from surface water in Kenya [33]. GII.17_2012 was also detected in Beijing before August 2014, causing sporadic cases of AGE. GII.17_2012 did not cause an increase in AGE outbreaks, nor did it replace the predominant GII.4 strains in these regions.

GII.17_2014 emerged in Beijing in August 2014. It replaced GII.4 Sydney_2012 as the predominant variant among AGE outbreaks in November 2014 as well as among sporadic cases of AGE after December 2014, causing an increase in the NoV activity in Beijing. In Jiangsu province, this strain was first found in October 2014, and it replaced GII.4 Sydney_2012 as the predominant variant in outbreaks after December 2014. GII.17_2004 dominated among sporadic cases since February 2015 in Jiangsu province, and a corresponding increase in the NoV detection rate among AGE cases was observed during this time [34]. In Guangdong province, GII.17_2014 was first detected in Guangzhou in November 2014, and it immediately replaced GII.4 Sydney_2012 as the predominant variant from November 2014 through January 2015, causing an increase in the number of NoV outbreaks [35]. The distribution of NoV genotypes among sporadic cases of AGE in Guangdong was not available for inclusion. In Japan, GII.17_2014 was a prevalent cause of NoV outbreaks from December 2014 onwards, becoming the predominant genotype in March 2015, but the total number of NoV outbreaks during this season was lower than that of previous years [13]. Based on the present data, it is probable that GII.17_2014 first appeared in August 2014 in Beijing, China, thereafter spread rapidly nationwide, and then expanded to Japan.

Amino acid changes were observed in the P2 domains of the VP1 protein between GII.17_2012 and GII.17_2014, which might result in the antigenic drift or altered receptor-binding properties [13, 34]. Additionally, differences were also found in the RdRp genes between GII.17_2012 and GII.17_2014, which could potentially result in the increased replication efficiency of GII.17_2014. The evolutionary history of GII.17_2014 remains unknown, and additional sequences of complete genomes from different times and regions should be analyzed. GII.17_2014 NoV was termed Kawasaki 2014 after the first near complete genome sequence (AB983218) submitted to GenBank [36]. However, this strain actually belongs to GII.17_2012 and was not the cause of the AGE outbreaks in China and Japan, so a new and uniform name is still needed.

Novel GII.17_2014 caused increased NoV activity in China, but a similar situation was not observed in Japan, even though this variant became the dominant genotype there in March 2015. In the temperate Northern hemisphere, NoV-associated AGE usually peaks during the winter months (December–February) [37]. GII.17_2014 emerged in China 2–4 months before this peak, whereas it appeared in Japan in December, when the previously dominant strain may have hindered the spread of this new variant. With the introduction of GII.17_2014 NoVs, an increase in the number of NoV outbreaks or the replacement of GII.4 Sydney 2012 viruses might happen outside of Asia, so monitoring the trends in the geographical spread and in the evolution of this variant is necessary. We recommend that public health departments prepare for a potential increase in NoV activity.

## Conclusions

A novel NoV GII.17 variant emerged in Beijing in August 2014, which then replaced the GII.4 variant Sydney_2012 as the predominant strain during winter 2014–2015 and caused an increase in NoV activity. A similar situation was also observed in Jiangsu, Guangdong, and Japan. This is the first time that a non-GII.4 genotype replaced the GII.4 variants as a predominant strain and caused increased cases and outbreaks of AGE. This change provides new challenges for the study of molecular evolution and the development of NoV vaccines.

## Abbreviations

AGE: acute gastroenteritis
GII.4: genogroup II genotype 4
NoV: norovirus
ORF: open reading frames
RdRp: RNA-dependent RNA polymerase
RT-PCR: reverse transcription polymerase chain reaction

## References

[1] Ramani S, Atmar RL, Estes MK (2014). Epidemiology of human noroviruses and updates on vaccine development. Curr Opin Gastroenterol.

[2] Hall AJ, Wikswo ME, Manikonda K, Roberts VA, Yoder JS, Gould LH (2013). Acute gastroenteritis surveillance through the National Outbreak Reporting System, United States. Emerg Infect Dis.

[3] Ahmed SM, Hall AJ, Robinson AE, Verhoef L, Premkumar P, Parashar UD (2014). Global prevalence of norovirus in cases of gastroenteritis: a systematic review and meta-analysis. Lancet Infect Dis.

[4] Teunis PF, Moe CL, Liu P, Miller SE, Lindesmith L, Baric RS (2008). Norwalk virus: how infectious is it?. J Med Virol.

[5] Glass RI, Parashar UD, Estes MK (2009). Norovirus gastroenteritis. N Engl J Med.

[6] Patel MM, Hall AJ, Vinjé J, Parashar UD (2009). Noroviruses: a comprehensive review. J Clin Virol.

[7] Green KY, Knipe DM, Howley PM (2013). Caliciviridae: the noroviruses. Fields virology.

[8] Thorne LG, Goodfellow IG (2014). Norovirus gene expression and replication. J Gen Virol.

[9] Bok K, Abente EJ, Realpe-Quintero M, Mitra T, Sosnovtsev SV, Kapikian AZ (2009). Evolutionary dynamics of GII.4 noroviruses over a 34-year period. J Virol.

[10] Trujillo AA, McCaustland KA, Zheng DP, Hadley LA, Vaughn G, Adams SM (2006). Use of TaqMan real-time reverse transcription-PCR for rapid detection, quantification, and typing of norovirus. J Clin Microbiol.

[11] Jiang X, Huang PW, Zhong WM, Farkas T, Cubitt DW, Matson DO (1999). Design and evaluation of a primer pair that detects both Norwalk- and Sapporo-like caliciviruses by RT-PCR. J Virol Methods.

[12] Kojima S, Kageyama T, Fukushi S, Hoshino FB, Shinohara M, Uchida K (2002). Genogroup-specific PCR primers for detection of Norwalk-like viruses. J Virol Methods.

[13] Matsushima Y, Ishikawa M, Shimizu T, Komane A, Kasuo S, Shinohara M (2015). Genetic analyses of GII.17 norovirus strains in diarrheal disease outbreaks from December 2014 to March 2015 in Japan reveal a novel polymerase sequence and amino acid substitutions in the capsid region. Euro Surveill.

[14] Noel JS, Fankhauser RL, Ando T, Monroe SS, Glass RI (1999). Identification of a distinct common strain of “Norwalk-like viruses” having a global distribution. J Infect Dis.

[15] Widdowson MA, Cramer EH, Hadley L, Bresee JS, Beard RS, Bulens SN (2004). Outbreaks of acute gastroenteritis on cruise ships and on land: identification of a predominant circulating strain of norovirus--United States, 2002. J Infect Dis.

[16] Bull RA, Tu ET, McIver CJ, Rawlinson WD, White PA (2006). Emergence of a new norovirus genotype II.4 variant associated with global outbreaks of gastroenteritis. J Clin Microbiol.

[17] Tu ET, Bull RA, Greening GE, Hewitt J, Lyon MJ, Marshall JA (2008). Epidemics of gastroenteritis during 2006 were associated with the spread of norovirus GII.4 variants 2006a and 2006b. Clin Infect Dis.

[18] Vega E, Barclay L, Gregoricus N, Williams K, Lee D, Vinje J (2011). Novel surveillance network for norovirus gastroenteritis outbreaks, United States. Emerg Infect Dis.

[19] van Beek J, Ambert-Balay K, Botteldoorn N, Eden JS, Fonager J, Hewitt J (2013). Indications for worldwide increased norovirus activity associated with emergence of a new variant of genotype II.4, late 2012. Euro Surveill.

[20] Hoa Tran TN, Trainor E, Nakagomi T, Cunliffe NA, Nakagomi O (2013). Molecular epidemiology of noroviruses associated with acute sporadic gastroenteritis in children: global distribution of genogroups, genotypes and GII.4 variants. J Clin Virol.

[21] Chan-It W, Thongprachum A, Khamrin P, Kobayashi M, Okitsu S, Mizuguchi M (2012). Emergence of a new norovirus GII.6 variant in Japan, 2008-2009. J Med Virol.

[22] Hoa-Tran TN, Nakagomi T, Sano D, Sherchand JB, Pandey BD, Cunliffe NA (2015). Molecular epidemiology of noroviruses detected in Nepalese children with acute diarrhea between 2005 and 2011: Increase and predominance of minor genotype GII.13. Infect Genet Evol.

[23] Nataraju SM, Pativada M, Chatterjee D, Nayak MK, Ganesh B, Bhattacharya MK (2011). Molecular epidemiology of norovirus infections in children and adults: sequence analysis of region C indicates genetic diversity of NVGII strains in Kolkata, India. Epidemiol Infect.

[24] Gao Z, Li X, Yan H, Li W, Jia L, Hu L (2015). Human calicivirus occurrence among outpatients with diarrhea in Beijing, China, between April 2011 and March 2013. J Med Virol.

[25] Kittigul L, Pombubpa K, Taweekate Y, Diraphat P, Sujirarat D, Khamrin P (2010). Norovirus GII-4 2006b variant circulating in patients with acute gastroenteritis in Thailand during a 2006-2007 study. J Med Virol.

[26] Galeano ME, Martinez M, Amarilla AA, Russomando G, Miagostovich MP, Parra GI (2013). Molecular epidemiology of norovirus strains in Paraguayan children during 2004-2005: description of a possible new GII.4 cluster. J Clin Virol.

[27] Mans J, Murray TY, Taylor MB (2014). Novel norovirus recombinants detected in South Africa. Virol J.

[28] de Andrade JS, Rocha MS, Carvalho-Costa FA, Fioretti JM, Xavier Mda P, Nunes ZM (2014). Noroviruses associated with outbreaks of acute gastroenteritis in the State of Rio Grande do Sul, Brazil, 2004-2011. J Clin Virol.

[29] Lee BR, Lee SG, Park JH, Kim KY, Ryu SR, Rhee OJ (2013). Norovirus contamination levels in ground water treatment systems used for food-catering facilities in South Korea. Viruses.

[30] Murray TY, Mans J, Taylor MB (2013). Human calicivirus diversity in wastewater in South Africa. J Appl Microbiol.

[31] Rajko-Nenow P, Waters A, Keaveney S, Flannery J, Tuite G, Coughlan S (2013). Norovirus genotypes present in oysters and in effluent from a wastewater treatment plant during the seasonal peak of infections in Ireland in 2010. Appl Environ Microbiol.

[32] Fernández MD, Torres C, Poma HR, Riviello-López G, Martínez LC, Cisterna DM (2012). Environmental surveillance of norovirus in Argentina revealed distinct viral diversity patterns, seasonality and spatio-temporal diffusion processes. Sci Total Environ.

[33] Kiulia NM, Mans J, Mwenda JM, Taylor MB. Norovirus GII.17 predominates in selected surface water sources in Kenya. Food Environ Virol. 2014; [Epub ahead of print] 10.1007/s12560-014-9160-6 25059212

[34] Fu J, Ai J, Jin M, Jiang C, Zhang J, Shi C (2015). Emergence of a new GII.17 norovirus variant in patients with acute gastroenteritis in Jiangsu, China, September 2014 to March 2015. Euro Surveill.

[35] Lu J, Sun L, Fang L, Yang F, Mo Y, Lao J (2015). Gastroenteritis outbreaks caused by norovirus GII.17, Guangdong Province, China, 2014–2015. Emerg Infect Dis.

[36] de Graaf M, van Beek J, Vennema H, Podkolzin AT, Hewitt J, Bucardo F (2015). Emergence of a novel GII.17 norovirus - End of the GII.4 era?. Euro Surveill.

[37] Ahmed SM, Lopman BA, Levy K (2013). A systematic review and meta-analysis of the global seasonality of norovirus. PLoS One.

